# Cross-Generational Differences in Independence and Interdependence: Discrepancies Between Their Actual and Ideal Selves in the Japanese Cultural Context

**DOI:** 10.3389/fpsyg.2021.676526

**Published:** 2021-06-15

**Authors:** Hirofumi Hashimoto

**Affiliations:** Graduate School of Literature and Human Sciences, Osaka City University, Osaka, Japan

**Keywords:** independence, interdependence, age, self-construal, cultural psychology, cultural agent, cultural game player

## Abstract

The current study examined cross-generational differences in both independent and interdependent self-construal. Two studies using samples from across Japan that included a wide age range demonstrated that, with increasing age, Japanese respondents scored higher on independence, which was measured by a self-expression scale, and lower on interdependence, as measured by a rejection avoidance scale. Furthermore, these cross-over effects were not observed with regard to participants' preferences (i.e., the ideal state of the self), but were only observed in their actual selves (i.e., the actual state of the self). These results suggest that the Japanese, especially younger generations, cannot help but behave in an interdependent way despite being eager to be independent.

## Introduction

The current Japanese cultural context is believed to be changing to a more independent and/or individualistic-oriented one (Hamamura, 2012; Beugelsdijk and Welzel, 2018; see also, Toivonen et al., 2011; Ogihara, 2017). If this is the case, younger Japanese people are expected to have more independent and less interdependent cultural values and beliefs than do older Japanese who have been exposed to more traditional cultural experiences. This expectation is based on the following two grounds: First, people who are motivated toward prevalent *cultural mandates* actively engage in cultural tasks, consciously or unconsciously (Kitayama et al., 2009; see also Kitayama and Uskul, 2011), and thus repeated engagement in cultural tasks over extended years is expected to lead older generations of Japanese people more strongly to culturally scripted psychological processes and behaviors. Second, younger generations are more extensively exposed to the Western culture of independence than older generations are. Therefore, younger Japanese people are expected to be more independent and less interdependent than older counterparts.

During the past 30 years, studies have demonstrated a broad range of cultural differences in cognitive, emotional, and motivational tendencies. According to the cultural psychological perspective, cultural differences in these psychological processes are a reflection of *cultural self-construal* (Markus and Kitayama, 1991, 2010; see also Cross et al., 2011, for a review) or *cultural mandates* (Kitayama et al., 2009), such as independence for North Americans and interdependence for East Asians. According to the standard assumptions among cultural psychologists, especially those who emphasize the influence of cultural self-construal, people in a culture actively engage in cultural tasks, either consciously or unconsciously, and repeated engagement in these tasks results in culturally scripted psychological processes and behaviors. From this perspective, culturally specific psychological processes would be expected to be more firmly engrained in older individuals who have had extensive exposure to these cultural practices than in younger individuals, who presumably have less cultural exposure. For example, people in an interdependent culture, such as Japan, are predicted to acquire interdependent psychological processes as they grow older. The standard assumption in cultural psychology emphasizes that there is a consistency between the individual psychological tendencies, such as personal preferences, internalized values or motivations, and the culturally shared meaning systems (Kitayama et al., 1997; Uchida et al., 2008). When it comes to a cycle of “mutually constituted culture and psychology,” which constitutes one of the core theories of cultural psychology (Shweder and Sullivan, 1993; Fiske et al., 1998), “the individual level often cannot be separated from the cultural level” (Markus and Kitayama, 1998, p.66); this view, which I term the *cultural agent view* of culture (see Hashimoto et al., 2011; Yamagishi, 2011), focuses on the consistency between culture-wide traits and individual psychology engrained through socialization process.

Although the traditional and standard assumption in cultural psychology is that people's thoughts and behaviors will be consistent with culturally shared self-construal (Markus and Kitayama, 1991, 2010) or cultural mandates (Kitayama et al., 2009), independence and interdependence are not always a matter of cultural traditions or standards. Yamagishi and colleagues proposed an alternative perspective that connects culturally shared beliefs with behaviors by arguing that humans are *cultural game players* who pursue their goals in anticipation of others' responses (Hashimoto et al., 2011; Yamagishi, 2011). The cultural game player view is based on the assumption that culturally specific behaviors are strategies, rather than simple expressions or personal preferences, tailored to social adaptive tasks that allow these players to acquire valuable resources from others (Yamagishi et al., 2008). According to this perspective, Hashimoto and Yamagishi (2015) posited that cultural differences exist in the strategies and *expectations* that promote the use of those strategies rather than *preferences*. They found that both Japanese people and Americans preferred independence to interdependence, without cultural differences. However, a strong cultural difference was found between American and Japanese people in their expectations regarding how others would judge both independent and interdependent people. As a result, a “preference-expectation reversal” emerged among the Japanese participants, in which they preferred independence to interdependence, but expected others would evaluate interdependence more positively than independence. This reversal was not observed among the American participants; they preferred independence to interdependence and expected others to as well. This preference-expectation reversal is also consistent with the findings of Hashimoto (2011), which indicated Japanese people who prefer to be independent express themselves, by default, as being less independent and more interdependent than they actually prefer. Hashimoto (2019) also empirically confirmed through observations of participants' behavior in public that Japanese university students prefer independence over interdependence; however, they expect others to be interdependent and identify themselves as interdependent in public. These findings suggest that Japanese people behave interdependently based on their expectations regarding the behavior of others rather than their own preferences.

From these two views (i.e., cultural agent and cultural game player), the current research conducted an exploratory examination of the cross-generational differences between the ideal and the reality of both independent and interdependent self-construal. To do so, the current study explores the differences, not with a sample of students, but with a wider age range of participants. To date, the question of how individuals' psychological processes, which might be consistent with their cultural self-construal, are acquired in a cultural context has not been sufficiently addressed. Therefore, the purpose of the present research is to examine generational differences in independence and interdependence among Japanese people. The secondary aim of this research is to clarify whether cross-generational patterns is observed with regard to participants' stated preferences (the ideal state of the self) and their actual selves (the actual state of the self). Based on the naïve assumptions of cultural psychology, it is possible to predict that Japanese people should acquire interdependent psychological process as they grow older and are exposed to more cultural experiences.

## Study 1: Cross-Generational Differences in Independence and Interdependence

Given that people in a culture get to think and behave naturally in a culture-specific way, as being suggested from the standard assumptions in cultural psychology or cultural agent view, Japanese interdependence should be scored higher with age. To examine this, Study 1 first attempted to figure out the generational differences in Japanese for independence and interdependence by using self-construal scales.

### Methods

The nationwide web-based survey was conducted after approval from the ethics committee of the author's affiliated university. Participants were self-selected from a pool of 4.65 million people, drawn from a marketing company called Cross Marketing CO (https://www.cross-m.co.jp/en/). The marketing company sent e-mail messages to potential participants across Japan and solicited their participation with monetary incentives and sampled participants avoiding any age group (the 20s, 30s, 40s, 50s, 60s) or gender bias. A total of 1,000 individuals (500 male and 500 female; age range 21–69, mean age = 44.69) were selected. Participants were first asked to provide their demographic characteristics, such as gender, age, prefecture of residence, and prefecture of birth. They were also asked to respond to self-construal scales.

Study 1 used the scales developed by Hashimoto and Yamagishi (2016), which are based on earlier scales administrated by Hashimoto and Yamagishi (2013). These scales separately measure two aspects of independence and interdependence, according to the argument that the core of cultural differences exists in the strategic aspects of independence and interdependence, within the contrast between *self-expression* and *rejection avoidance*. More specifically, Hashimoto and Yamagishi (2013, 2016) argue that self-expression is a strategy used to make oneself predictable to others. They further contend that in long-term and “closed” relationships, where acceptance from closely related providers of needed resources is the primary condition for survival, being sensitive to these resource providers' feelings and avoiding their rejection (which the researchers called rejection avoidance) is an adaptive strategy. Hashimoto and Yamagishi (2013) conducted a cross-cultural study using the scales they constructed to measure these two types of cultural beliefs and found that Japanese people and Americans showed cultural differences in the contrast between self-expression and rejection avoidance. Hashimoto and Yamagishi (2016) then revised the independent and interdependent scales and confirmed the robustness of their previous findings (Hashimoto and Yamagishi, 2013). Owing to the need to reduce the number of items as much as possible, Study 1 used only part of Hashimoto and Yamagishi's 2016 scales, the self-expression and rejection-avoidance subscales, each consisting of five items (response options ranged from 1 = “does not describe me at all” to 7 = “describes me very well”). For example, the participants were asked to choose how well the following statements described them: “I always express my opinions in a straightforward manner” (a self-expression item) or “I find myself being concerned about what others think of me” (a rejection-avoidance item).

### Results

**Figure 1:**
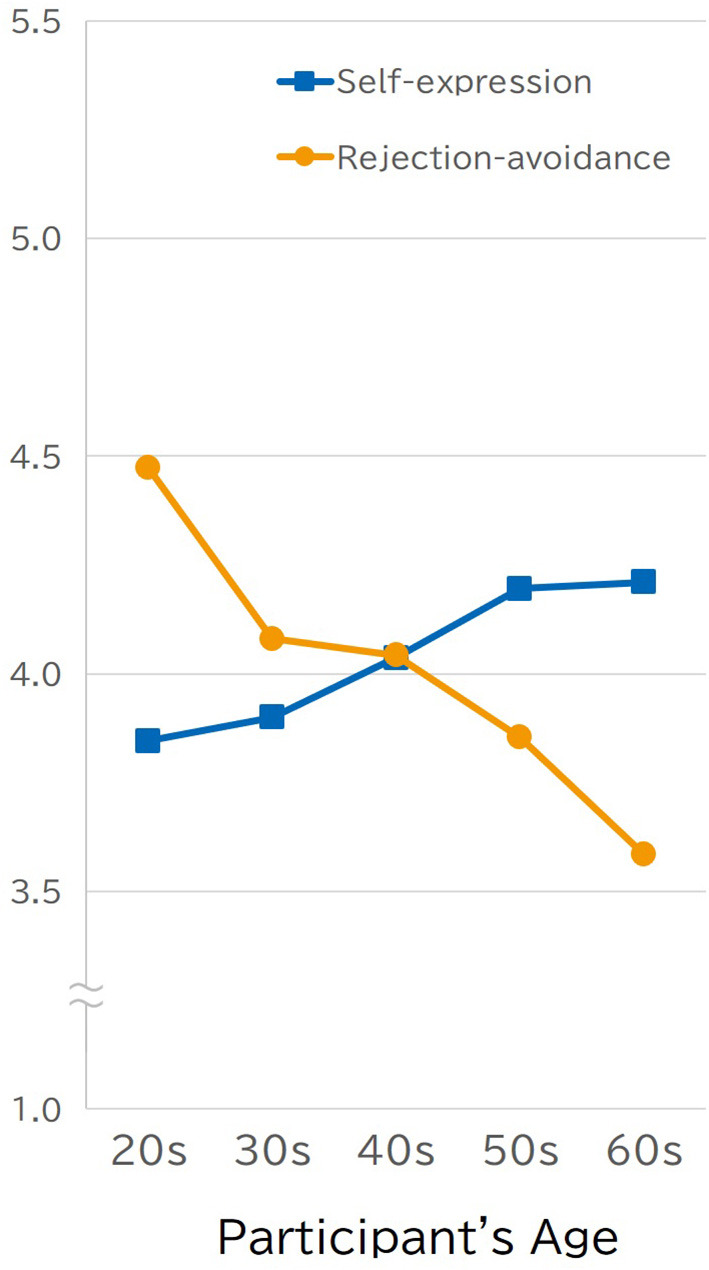
Positive correlation between the scores of self-expression and the respondents' age and negative correlation between the rejection-avoidance and the respondents' age (Study 1).

A confirmatory factor analysis was conducted to test the adequacy of fit of the two-factor model for the self-construal subscales. The goodness-of-fit indices were within an acceptable range, but was not so much as expected (CFI = 0.901, RMSEA = 0.114, GFI = 0.907); therefore, two subscales, “self-expression” (α = 0.841) and “rejection avoidance” (α = 0.863), were used for analysis. The self-expression subscale (*M* = 4.04, *SD* = 1.04) was negatively correlated with the rejection avoidance subscale (*M* = 4.01, *SD* = 1.18; *r* = −0.23, *p* < 0.0001). As shown in Figure 1, as age increased, participants scored higher on independence, as measured by the self-expression subscale (*r* = 0.13, *p* < 0.0001), and lower on the interdependence, as measured by the rejection-avoidance subscale (*r* = −0.24, *p* < 0.0001). Given that self-expression and rejection avoidance are considered to be the core aspects of interdependent and independent self-construal, respectively, these findings in Study 1 should be given special attention.

## Study 2: Generational Differences in the Ideal and Actual Self

The generational differences observed in Study 1, regarding the participants' responses to the self-construal scales which to be found that there existed cultural differences (Hashimoto and Yamagishi, 2013, 2016), did not support the cultural agent view. The first goal of Study 2 was to replicate the pattern of generational differences observed in Study 1. To do so, Study 2 included another aspect of interdependence: *harmony-seeking*. Study 1 narrowly focused on rejection avoidance as an aspect of interdependence; however, rejection avoidance differs from harmony-seeking, which is commonly assumed in cultural psychology to be related to interdependence. Harmony-seeking is a strategy used to promote cooperative relationships with others; promotion of mutual cooperation is a fundamental condition for human society, regardless of culture (Hashimoto and Yamagishi, 2016; see also, Yamagishi and Hashimoto, 2016). Study 2 explore how the harmony-seeking aspect relate to age.

The second goal of Study 2 was to provide support for the cultural game player view by confirming the additional prediction that the generational pattern obtained in Study 1 would not be observed with regard to the participants' preferences (the ideal state of the self) and would only be observed in their actual selves (the actual state of the self). For these purposes, participants were asked to respond to three subscales of Hashimoto and Yamagishi's self-construal scale in two different contexts. First, participants were asked to indicate how well each of the scale items described the way that they are or the way that they behave; this is the standard context commonly used in self-construal questionnaire studies. According to the cultural agent view, how people feel, think, and behave is based on their preferences (e.g., Kim and Markus, 1999). However, according to the cultural game player view, people's feelings, thoughts, and behaviors are a compromise between their preferences and the anticipated consequences of feeling, thinking, and behaving in a particular way (e.g., Hashimoto et al., 2011). Based on these two views, participants were asked to respond to the same set of questions twice, both as how they would actually answer and from the point of view of the ideal self (i.e., respond as if they were the type of person they wanted to be). In this way, I measured their preferences through images of the ideal self's responses. I predicted that generational differences (as well as cultural differences) are based on the way that people adaptively address social reality (i.e., the actual self) and not their preferences (i.e., the ideal self that they want to be).

### Methods

As in Study 1, a web-based survey was conducted after approval from the ethics committee of the author's affiliated university. The survey agency, Cross Marketing (https://www.cross-m.co.jp/en/), sent e-mail messages to potential participants across Japan and solicited their participation with monetary incentives. The participants were sampled to avoid any age group (20s, 30s, 40s, 50s, 60s) or gender bias. A total of 500 individuals (250 male and 250 female, ages 20–69, mean age = 44.81) were first asked to provide demographic details, such as their gender, age, prefecture of residence, and prefecture of birth. They were also asked to complete the self-construal scales including the harmony seeking scale. I used the same set of the three self-construal scales to measure the actual self and ideal self. Participants were first asked to rate how well each item on the three scales described the way that they are and how they behave. After completing the three scales, they were also asked to rate the same set of question items from the point of view of their ideal self (i.e., the person who they wanted to be).

### Results

Similar to Study 1, confirmatory factor analyses were conducted to test the adequacy of fit of the three-factor model for the self-construal subscales. The goodness-of-fit indices were acceptable for the actual-self scales (CFI = 0.902, RMSEA = 0.089, GFI = 0.893) and ideal-self scales (CFI = 0.905, RMSEA = 0.098, GFI = 0.880). The reliability of the actual-self scales was α = 0.832, 0.861, and 0.854, for self-expression, harmony-seeking, and rejection avoidance, respectively. The reliability of the ideal-self scales was α = 0.870, 0.891, and 0.878, respectively.

**Figure 2:**
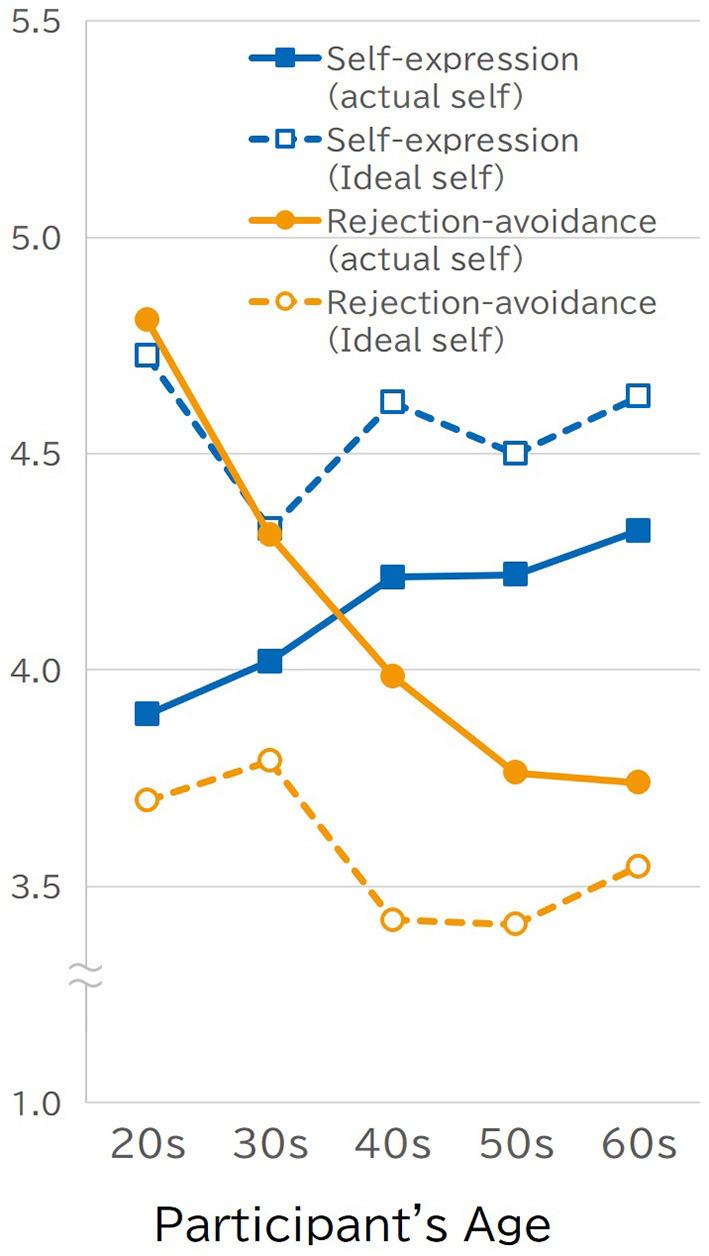
Relationships between the participants' actual-self and ideal self scores on the self-expression/rejection-avoidance scales and their age (Study 2).

Figure 2 shows the participants' actual-self scores on the self-expression/rejection-avoidance scales and their age; the results of Study 1 were replicated in Study 2. The consistency in the cross-generational pattern shown in Figures 1 and 2 indicates the robustness of the pattern identified in Study 1. This suggests that Japanese people become more independent (i.e., self-expression) and less interdependent (e.g., rejection-avoidance) as they age. Participants' age was positively correlated with their self-expression scores (*r* = 0.16, *p* < 0.0001) and negatively correlated with rejection avoidance scores (*r* = −0.34, *p* < 0.0001). The pattern shown in Figure 2 also provides clear support for the validity of the cultural game player view. The ideal self's responses to the self-expression scale (*r* = 0.00) did not correlate, and rejection avoidance (*r* = −0.09) was slightly negatively correlated with age. Regardless of their age, respondents considered their ideal self to be an independent person, not an interdependent person who avoided rejection. These differential effects of age on the actual and ideal selves generated the reversal found between the actual and the ideal selves among young participants, which was similar to the preference-expectation reversal (Hashimoto and Yamagishi, 2015). Participants in their 20s preferred an independent person who engaged in self-expression to an interdependent person who was concerned with acceptance from others; however, they reported they actually have more in common with the latter than the former. Those who were in their 40s, 50s, and 60s preferred to be independent over interdependent, similar to their younger counterparts; however, also they reported that how they actually felt, thought, and behaved were in accordance with their preferences.

**Figure 3:**
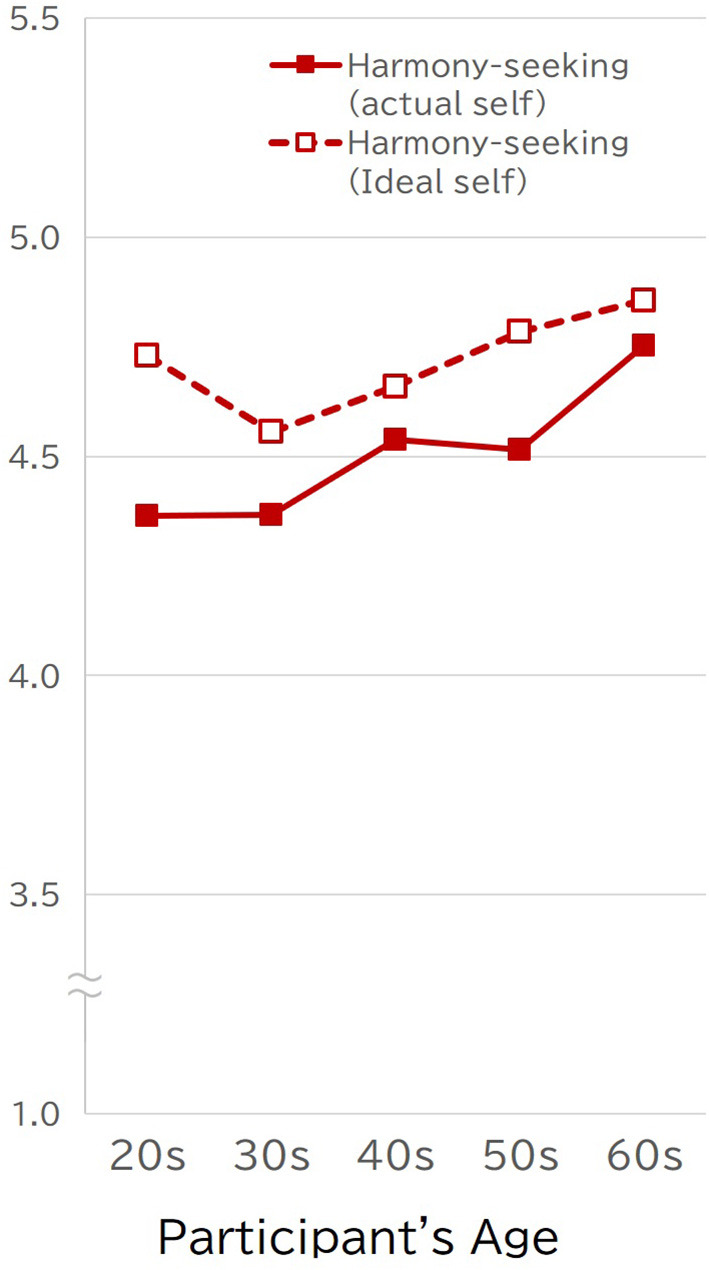
Actual and ideal self scores on the harmony-seeking scale across respondents' age (Study 2).

The pattern shown in Figure 3 provides partial support for the cultural agent view. Participant age was positively correlated with their harmony-seeking scores (*r* = 0.14, *p* < 0.0001); however, their preferences for harmony-seeking did not increase with age (*r* = 0.06).

## General Discussion

In Study 1, I focused on cross-generational differences in the contrast between self-expression and rejection avoidance, which have already been confirmed to show differences between Japan and the United States (Hashimoto and Yamagishi, 2016). The current results showed that self-expression tended to increase with age, while rejection avoidance decreased. In Study 2, these effects of age were replicated, and it was also shown that the effects exist in relation to the actual self, but not the ideal self. Given that self-expression and rejection avoidance are considered to be core aspects of independent and interdependent self-construal, respectively, these findings were difficult to explain from a cultural agent view^1^.

The current findings showed that the Japanese ideal self was independent regardless of their age, and the discrepancies between their actual self and ideal self on independence were negatively correlated with their age, which suggests that independence and interdependence are not simply a matter of cultural traditions or mandates. They are *ways of life* to which each generation in every society adapts with regard to the social situations they face. How people think and behave is inseparably intertwined with the type of social environments they live in. Facing the tidal wave of globalization and cultural Westernization, it is widely believed that new generations of East Asians are becoming more independent and less interdependent. The current study disconfirms this naïve view of cultural change, at least with regard to the independence of the Japanese respondents. Contrary to common belief, younger Japanese respondents are found to be less independent than older ones, suggesting that Japanese, especially younger generations, cannot help but behave in an interdependent way despite being eager to be independent.

One potential interpretation regarding the discrepancies between their actual and ideal selves is that the Japanese youth who are facing the increasingly insecure labor market and declining security due to the worsening national pension system are more strongly motivated to seek secure jobs and to stick with those jobs once they obtain them. This makes them make likely to follow the perceived expectations of others around them. Furthermore, it would be likely that with age, individuals accumulate social and economic resources and become less dependent on a particular relationship or group for their survival; the accumulation of social and economic resources relieves older Japanese generations from being dependent on limited resource providers and allows them to behave more independently. As a consequence, younger generations may be less independent than older generations. However, another interpretation would be possible: the reversal is caused by a “cultural lag” (Triandis, 1994) that is specific to times of cultural transition. Individuals' preferences are generally more susceptible to new values and ideas, whereas their beliefs about others' values and preferences (that is, expectations) take more time to change. Given the current cultural transition that is accompanied by globalization, it is possible that Japanese people who are exposed to the Western culture of independence are rapidly changing their preferences from the traditional, interdependence-oriented ones to the more Westernized, independence-oriented preferences. However, their views that other people and institutions are still acting on traditional values may be slower to change. This differential speed of change in regard to preferences and expectations may constitute the foundation for the discrepancies between their actual and ideal selves. Needless to say, these interpretations are not based on the specific data at the current stage, therefore, future researches should examine more carefully why the discrepancies occurs.

There are limitations that need to be addressed. One issue is that the current research only showed correlational studies; hence, the arguments based only on the correlational analyses should be used with precaution. Although the findings could demonstrate generational differences of Japanese independence and interdependence, clues are not provided as to why such generational differences were observed. It should be noted that both data from large-scale international comparative studies and longitudinal studies (e.g., McCrae et al., 2005; Terracciano et al., 2005) will help clarify some of the questions addressed in this study, such as the universality of increased independence and decreased interdependence with age. In terms of the cultural models of selfhood, data from large-scale international comparative studies have already been demonstrated (Vignoles et al., 2016). Future studies analyzing the cultural, gender, and age differences at the same time may have more implications. It should also be noted that the current study was not the first to find that older Japanese people are more independent and less interdependent than younger Japanese people. In an earlier study, Takata (1999; Study 2) reported a similar finding, although the report was published in Japanese and not widely noticed or shared outside of Japan. In that study, using the independent and interdependent self-construal scales he constructed, Takata found that among the 11,382 participants surveyed, older Japanese people were more independent and less interdependent than younger Japanese people. Many of the items included in Takata's interdependence scale are similar to those on the rejection avoidance scale used in the present study. Thus, the finding that older Japanese exhibit less interdependence seems to be robust among Japanese people. Finally, it is also problematic that the data of the current research were cross-sectional, therefore, the current results could not attribute the generational differences to the effects of age itself, generation-specific experiences, or general historical trends. Considering more the historicity of the cultural self-construal would be needed in future studies.

## Data Availability Statement

The raw data supporting the conclusions of this article will be made available by the authors, without undue reservation.

## Ethics Statement

The studies involving human participants were reviewed and approved by the Ethics Committee at Yasuda Women's University. Written informed consent was not provided because the survey agency, Cross Marketing CO. (https://www.cross-m.co.jp/en/), sent e-mail messages to potential participants, and encouraged their participation with monetary incentives. Those who did not agree to participate were unable to do so. Therefore, I considered that all participants agreed to participate in my survey.

## Author Contributions

The author confirms sole responsibility for the following: study design, data collection, analysis and interpretation of results, and manuscript preparation.

## Conflict of Interest

The author declares that the research was conducted in the absence of any commercial or financial relationships that could be construed as a potential conflict of interest.

## Footnotes

^1^However, by narrowing the focus to harmony-seeking, not rejection avoidance, as a core aspect of interdependence, it might be possible to consider the current results as demonstrating that Japanese “interdependence” increases with age. Even though, it should be noted that earlier studies found no expected cultural differences between people from Japan and North America in relation to harmony-seeking aspects of interdependence (Hashimoto and Yamagishi, 2013, 2016; see also, Ishii and Eisen, 2018; Eisen and Ishii, 2019). Furthermore, older generations are known to be more cooperative with others (Matsumoto et al., 2016), even in Western cultures (Van Lange et al., 1997). Therefore, the increase in the score of harmony-seeking with age found in Study 2 may be a universal pattern rather than a culturally specific one. However, due to the relative paucity of research in which these two types of interdependence are separately analyzed, it would be premature at this point to draw any firm conclusions on this issue.
